# Microfluidic Microalgae System: A Review

**DOI:** 10.3390/molecules27061910

**Published:** 2022-03-15

**Authors:** Anand Baby Alias, Shubhanvit Mishra, Gaurav Pendharkar, Chi-Shuo Chen, Cheng-Hsien Liu, Yi-Ju Liu, Da-Jeng Yao

**Affiliations:** 1 Institute of NanoEngineering and MicroSystems, National Tsing Hua University, Hsinchu 30013, Taiwan; alias.anand@gmail.com (A.B.A.); shubhanvit@gmail.com (S.M.); liuch@pme.nthu.edu.tw (C.-H.L.); 2 Department of Power Mechanical Engineering, National Tsing Hua University, Hsinchu 30013, Taiwan; g.pendharkar@ieee.org; 3 Department of Biomedical Engineering and Environmental Sciences, National Tsing Hua University, Hsinchu 30013, Taiwan; chen.chishuo@gmail.com; 4 Food Industry Research and Development Institute, Hsinchu 300193, Taiwan; lyj20@firdi.org.tw

**Keywords:** microalgae, microfluidic, photobioreactor

## Abstract

Microalgae that have recently captivated interest worldwide are a great source of renewable, sustainable and economical biofuels. The extensive potential application in the renewable energy, biopharmaceutical and nutraceutical industries have made them necessary resources for green energy. Microalgae can substitute liquid fossil fuels based on cost, renewability and environmental concern. Microfluidic-based systems outperform their competitors by executing many functions, such as sorting and analysing small volumes of samples (nanolitre to picolitre) with better sensitivities. In this review, we consider the developing uses of microfluidic technology on microalgal processes such as cell sorting, cultivation, harvesting and applications in biofuels and biosensing.

## 1. Introduction

Algae are photosynthetic organisms that grow in lakes, ponds, rivers, oceans and even wastewater. Algae can broadly be classified as *Rhodophyta* (red algae), *Phaeophyta* (brown algae) and *Chlorophyta* (green algae), and are classified by size as macroalgae or microalgae. Macroalgae constitute a large, plant-like form of algae, of which seaweed is a type. Microalgae are single microscopic cells and might be prokaryotic, such as cyanobacteria (*Chloroxybacteria*), or eukaryotic, similar to green algae (*Chlorophyta*). Carbon compounds found in microalgae can be used in biofuels, health supplements, medicines, and cosmetics [1]. Microalgae are established to be useful as a renewable and sustainable feedstock for various applications, including biofuel, smart nutrition, biopharmaceuticals, cosmeceuticals, biosensing and space technology. These organisms accumulate many functional biochemical components: pigments and carotenoids derived from proteins, carbohydrates and lipid groups. Microalgal biomass, which might be used for multivalorisation in biorefinery settings, allows for the development of biofuels over a wide range and other valuable biotechnological products with a large added value. Microalgal research is currently conducted among a few species [2,3]. The possibility of achieving a commercially viable microalgal biofuel production can improve the productivity in a proposed local environment by bioprospecting better-performing microalgal strains with greater stress tolerance [4,5,6,7,8]. In particular geographical locations, microalgal strains have been discovered in the local environments in which their production exhibits superior adaptability to their local environmental conditions [7,9]. As microalgae grow in a natural environment, native microalgae serve as indicators for various environmental monitoring applications. With metabolic and genetic engineering, as well as through directed mutation, it is possible to develop further strains that are highly efficient in microalgal production that effectively produce required high-value products, have phenotypes that are similar to local strains, and possess tolerance against abiotic and biotic stress such as temperature, salinity and pathogens that might be encountered in the local environment [6,8].

Microfluidics, which began in the 1980s, have been used to research focal points since then. Microfluidics is a field that treats the study of fluids at a microliter level [10]. Various biomedical approaches of microfluidic devices are explained elsewhere [11,12,13]. Other microfluidic applications in microorganisms and microalgae research, such as monitoring of microorganisms using dielectrophoretic (DEP) microfluidic chips [14] or microbial bioenergy [15], are reported in various journals. The use of cutting-edge techniques is necessary, however, to maximize the production, quality and the economic elements of both upstream and downstream operations. Because of the small size and dilute microalgae culture, the utilization of microfluidic-based devices for both fundamental research and industrial applications of microalgae is hence becoming increasingly important. Because of their capacity to perform numerous activities, microfluidic-based devices outperform their competitors in sorting and analysing small volumes of samples (ranging from nanolitres to picolitres) with high sensitivity. Microfluidic-based devices outperform their competitors in terms of their ability to perform multiple functions, such as simulating natural environments of living cells, portability and the ability to control and to analyse samples in small amounts (from nanolitres to picolitres), even with a single cell on a chip, with great sensitivity and accuracy. 

## 2. Cell Identification by Identification of Cell Property or Strain Selection or Species

The existence of over one million distinct species of microalgae in the world [16] is believed to be accurate; their identification is crucial for diverse purposes, including environmental monitoring, public health issues and biofuel production. Microalgae species of a modest number are studied previously and currently, although many species are present. A spectrum of micro-algal strains is also restricted to the local environment; these strains are superior to global species because of their adaptability to local environmental circumstances, which makes them superior to global species [7,9]. 

The most essential phase of cell identification is to select highly resistant microalgal strains, which must be conducted with care to avoid contamination during large-scale cultivation to achieve high quality and lipids in huge quantities.

Diverse applications, ranging from diagnosis to environmental monitoring, have been demonstrated using chemical and biological detection, which will lead to the development of strains capable of efficiently generating high-value products after they have been identified using genetic and metabolic engineering techniques. They might also exhibit characteristics that native strains lack, such as abiotic and biotic stress, resulting from local environmental conditions [4,8].

Molecular biology is an effective method to identify microalgae in which the algal specimens are taken to a laboratory from diverse environments and examined using the image, and later genotyped. This procedure is tedious, with high labour work, but reliable. Flow cytometry has been utilized to automate and accelerate this procedure in recent years [17,18], but the high cost of such devices is a mitigating factor. Furthermore, because these approaches rely on large equipment, analysis of the collected samples on site is impracticable; bioprospecting materials must hence be evaluated after that work in a laboratory setting. Microfluidic flow cytometry has the potential to be a viable alternative technology because of its compactness and portability, which enables microalgae analysis and classification on site during bioprospecting. Benazzi et al. [19] developed the first microfluidic cell cytometer that accomplished the monitoring of chlorophyll autofluorescence of cells flowing across an optical detection zone in a microfluidic channel, which was capable of analysing and distinguishing microalgal cells. An impedance–spectroscopy electrode was included in the flow channel to estimate cell sizes, allowing simultaneous analysis of impedance signal and autofluorescence of cells flowing through the chip’s detecting zone. Later, Hashemi et al. [20] developed an improved microflow cytometer on integrating the two-dimensional flow focusing by the usage of flow-guiding grooves at the bottom and a top microchannel that enabled the sample flow to become concentrated in both horizontal and vertical directions, resulting in two symmetric streams that are wrapped through central microalgae-laden sample streams. The side scattering and chlorophyll autofluorescence characteristics of three various microalgae species were examined using laser excitation at 488 nm, demonstrating increased detection capabilities of samples of diameter 1 μm, although the laser at 488 nm utilized in this system did not strongly excite chlorophyll a. As a result, they used two lasers for excitation, at 404 and 532 nm, which were nearer the maximal excitation wavelengths of chlorophyll and phycoerythrin [21]. This microsystem distinguished four microalgal species, ranging from 1–50 μm and with varied quantities of intrinsic chlorophyll and phycoerythrin.

Along with the flowcytometry principles in microfluidic systems, numerous laser sources, detectors and filters for wavelength selection are required, which makes shrinking the size and cost of integrated microfluidic flow cytometry systems challenging. Schaap et al. [22,23] created an optofluidic system that could differentiate microalgae species with a simplified configuration. Microalgae inside the microchannel were illuminated with laser light guided through a curved waveguide, which was perpendicular to the next microfluidic channel. The distinctive wavelets associated with cell geometry and size were detected with a single laser source and a single quadrant-cell photodetector.

### 2.1. Microfluidic Flow Cytometry for Cell Characterization

Microscopy combined with biological study has been the age-old method of identifying microalgae. Whereas it offers ease of sample preparation, imaging and analysis followed by genotyping, it has several disadvantages such as extended duration and, most crucially, being intensive of labour and prone to manual error. Flow cytometry has been the preferred method for two decades with several advantages over traditional methods [17,24], but substantial costs of instrument, bulky instruments and thereby lack of measurement capability on site make it the least preferable choice.

Microfluidic technology has paved the way in recent years, making a portable, small and compact-sized device that enables microalgae analysis in house, leading to a classification in the course of bioprospecting.

Benazzi and colleagues developed a microfluidic cytometer with detection electrodes 20 µm wide and 20 µm apart and patterned at the bottom microchannel to differentiate and to study algal cells in the laboratory. Using the notion of varying impedance signals, this method becomes applicable such that the impedance changes that occur because of cell parameters such as permittivity, cell cytoplasm, membrane capacitance, cell size and conductivity serve to determine the impedance change [25].

Hashemi et al. [26] constructed a microflow cytometer. Their design differs from others, with a chevron-shaped trench on the top and bottom of a flow channel. The grooves produce two sheath streams on both sides of the sample stream to envelop a central core stream. Light at two wavelengths from lasers was shone on the sample; the scattered-light and fluorescence signals were detected. 

Schaap et al. created a glass microchip with a laser source and a waveguide to illuminate the entire channel height. With images taken by the camera, the intensity of the detected light was recorded. The photodiode signals of nine algae species were discovered to be distinct, and five mixed species were identified using neural-network classification [15,27,28,29].

**Figure 1:**
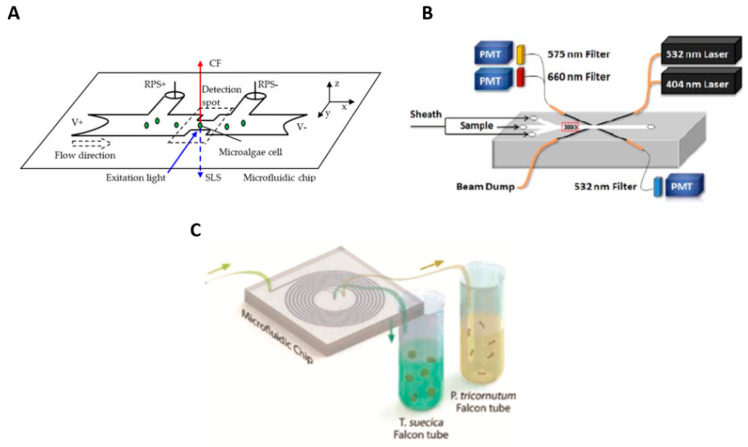
(**A**) Illustration of the principle of classification of single microalgae cells based on the simultaneous detection of CF (chlorophyll fluorescence), side light scattering (SLS), and resistance pulse-sensing (RPS) signals. Reproduced with permission from [18] J. Wang et al., “A new microfluidic device for classification of microalgae cells based on simultaneous analysis of chlorophyll fluorescence, side light scattering, resistance pulse sensing”, Micromachines, 2016. (**B**) Microfluidic devices for microalgae strain identification; optofluidic characterization of marine microalgae using a microflow cytometer. Reproduced with permission from [21] N. Hashemi et al., “Optofluidic characterization of marine algae using a microflow cytometer”, Biomicrofluidics, 2011. (**C**) A spiral-shaped microfluidic channel can separate an oleaginous microalga. Reproduced with permission from [31] M. S. Syed et al., “Selective separation of microalgae cells using inertial microfluidics”, Bioresource technology, 2018.

Lipid accumulation with photosynthetic characteristics can be assessed together with a microfluidic cytometer [30]. A 2D hydrodynamic focusing microchip obtained the induction curves from chlorophyll fluorescence to determine a photosynthetic quantum yield. The results showed that the lipid content inside the nutrition-limited algal cells is inversely correlated with quantum efficiency. The various microfluidic flow cytometry approaches are depicted in Figure 1.

### 2.2. Analysis of Cell Viability

One way to determine cell viability is through the analysis of cellular capacitance. Living cells have an intact plasma membrane, whereas dead cells have damaged ones. These damaged membranes might alter the dielectric characteristics of cells, which can be detected on examining the capacitance of the cells.

Song et al. proposed a three-dimensional microfluidic capacitive sensor [32]; the microfluidic chip was characterized with *Dunaliella salina* cells. A change in the capacitance detected the live and dead cells. Being dependent on frequency, the capacitance change is greater for live cells than for dead cells.

Another way to analyse the viability of microalgae cells using a microfluidic platform is to detect the intensity of chlorophyll fluorescence, which is proportional to the chlorophyll content inside the microalgae and which can be used to evaluate the photosynthetic capacity and activity of the cells. Wang et al. designed a microfluidic platform consisting of a laser diode to illuminate the samples based on this idea; the conversion of the chlorophyll autofluorescence intensity sensed with a photodiode into a matching output voltage is possible [33]. 

**Figure 2:**
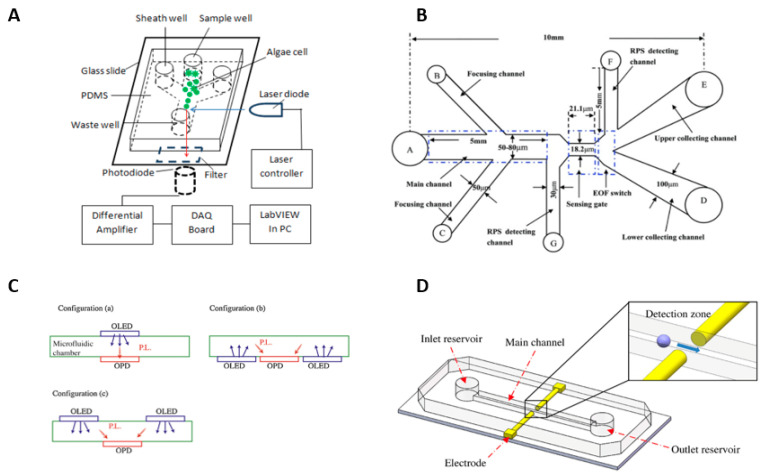
Microalgae viability analysis. (**A**) The schematic diagram of the chlorophyll fluorescence detection system. Reproduced with permission from [30] J. Wang et al., “A label-free microfluidic biosensor for activity detection of single microalgae cells based on chlorophyll fluorescence”, Sensors, 2013. (**B**) A schematic diagram of the system setup of resistive pulse sensor (RPS)-based particle detection and sorting [34] Y. Song et al., “Automatic particle detection and sorting in an electrokinetic microfluidic chip”, Electrophoresis, 2013. (**C**) Different OLED/OPD layouts for lab-chip fluorescence sensor. Reproduced with permission from [35] F. Lefèvre et al., “Integration of fluorescence sensors using organic optoelectronic components for microfluidic platform”, Sensors and Actuators B: Chemical, 2015. (**D**) Schematic diagram of the chip structure and the capacitive detection zone. Reproduced with permission from [32] Y. Song et al., “Capacitive detection of living microalgae in a microfluidic chip”, Sensors and Actuators B: Chemical, 2014.

A small microfluidic setup with sample transport controlled by electrokinetic flow was demonstrated [34]. This microfluidic device can assess cell viability in response to various chemical treatments. Later, to assess *Chlamydomonas reinhardtii* cell populations, a microfluidic chip combined with an organic light-emitting diode (OLED) and an organic photodetector (OPD) was developed [35]. These OLED and OPD were successful in decreasing device costs while also attaining portability. A blue OLED, an OPD with a significant quantum efficiency in the wavelength region 680–700 nm, and absorption filters with large transmittance and attenuation, with greater than 80% and 40 dB, respectively, were developed in this work. Figure 2 represents the various microalgae viability analysis.

Algae-based biosensors have shown the ability to detect analysts of agro-environmental and security concern in a sensitive, long-term, and multiplexed manner. Their benefits include the availability of various algal bioreceptors, including entire cells and photosynthetic subcomponents, the capacity to incorporate them into tiny systems, and the ability to monitor the environment continuously. By utilizing promising nanomaterials and smart materials, we are improving the analytical performance of such sensors in terms of selectivity and stability using nanotechnology and materials science [36].

The study of photosynthetic pesticides in river water was achieved using a highly sensitive algal biosensor. This technology demonstrated carbon black’s ability to detect and monitor changes in algal oxygen evolution during the photosynthetic process in the presence of pollutants [37].

Based on *Chlamydomonas reinhardtii* complete cells immobilized on paper-based screen-printed electrodes modified with carbon black nanomaterials, a dual electro-optical biosensor for herbicide monitoring was developed. In terms of sensitivity, reproducibility, and working and storage stability, the biosensor outperforms other photosynthetic biosensing devices now available in the literature [38].

## 3. Microfluidic-Based Microalgal Sorting

The phenomenon of dielectrophoresis was introduced by Pohl et al. [39,40], defining the basic DEP formula that determines cell movement under the DEP force, attempting continuous separation of *Chlorella vulgaris*, and identifying a cell movement reliance on media salinity in early work. Early researchers discussed work related to the dielectric spectral analysis of cells [40,41,42,43], essential system development and accompanying electrode designs [44,45].

In comparison to other cells, an exciting and differentiating characteristic of microalgae is their ability to amass large amounts of internal lipids [46], making them attractive for cell manipulation with DEP. The resulting DEP force can be variable, making it suitable for use for DEP-based separation and characterization, among other applications. Because a lipid has dielectric properties distinct from those of normal cytoplasm, microalgae can be distinguished by their size as a signal of their characteristics or changes in physiology, creating yet another possibility for DEP-based separation. In this review, DEP microfluidic devices for microalgae applications are critically examined from two perspectives: first, from the perspective of the microfluidic structures that have been used, and second, from the perspective of the applications that have been made possible by such DEP microfluidic devices [47,48].

### 3.1. Electric Field-Based Sorting

Cell sorting has been an essential step to separate cells based on their properties. Microalgae sorting is typically undertaken to separate them from a large sample population for various applications based on their properties such as size, viability, lipid content, growth rate, pigment content, tolerance to environmental factors, etc. [49].

Among various methods, the use of an electric field for sorting has been a widely adopted method. A non-uniform electric field leads to a dielectrophoretic force that is related to the dielectric properties of cells and the medium in which they are present [50,51,52].

Deng et al. [53] proposed a microfluidic device to separate microalgae with varied lipid content by dielectrophoresis (DEP). The exposure of microalgae to a non-uniform AC electric field leads to either a positive DEP (pDEP) or a negative DEP (nDEP) based on their lipid content, which moves cells either towards the electrodes (positive DEP) or away from the electrodes (negative DEP). A microfluidic chip to sort C. Vulgaris cells based on lipid content has been demonstrated with efficiencies 11% and 45%. Although the cell sorting capability was well demonstrated, the entire device throughput was minute, as the separation occurred only without flow. An improved design with high throughput and continuous cell sorting, related to the relative strength of hydrodynamic forces and the opposing DEP forces, influences the microalgal cells flowing through the device [54]. All cells experienced a hydrodynamic force of the same kind with varied magnitude of opposing DEP force depending on their lipid content, resulting in varied flow trajectories along the microchannel. In an interdigitated electrode design, a positive DEP force is more vital than an opposing DEP force [55,56,57]. A continuous-flow cell-sorting system capable of sorting cells based on their lipid content was developed [58]. At frequency 50 MHz, cell DEP response is predominantly determined by the dielectric characteristics of the cytoplasm, which are regulated by the quantity of lipid accumulated within cells. There was a significant split between high- and low-lipid accumulating *C. reinhardtii* mutants, with low-lipid cells experiencing a positive DEP force and exhibiting a zigzag trajectory. In contrast, high-lipid cells moved along the hydrodynamic stream through the influence of opposing DEP forces.

Another microfluidic-based platform called direct-current dielectrophoresis (DC-DEP) has been proposed; it uses insulating microstructures such as pillars or hurdles in a microchannel to generate a spatially defined non-uniform electric field from a uniform DC electric field rather than an AC field generally required in DEP systems [59]. With various benefits compared to AC–DEP systems, such as simple microfabrication, decreased biofouling of the test region and higher throughput, the DC–DEP has proven to help analyse microalgae properties such as viability and size and separating cells.

**Figure 3:**
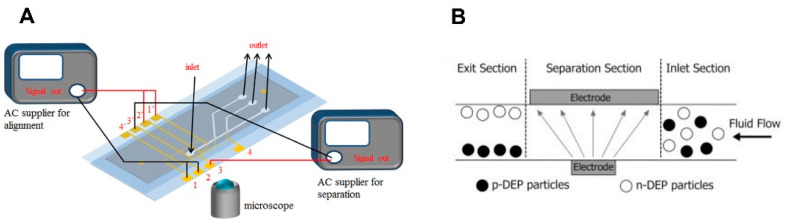
(**A**) Schematic of the experimental setup for separation of microalgae with different lipid contents. Reproduced with permission from [53] Y.-L. Deng et al., “Development of flow through dielectrophoresis microfluidic chips for biofuel production: Sorting and detection of microalgae with different lipid contents”, Biomicrofluidics, 2014. (**B**) Schematic drawing of DEP-based microfluidics devices: non-uniform electric field by means of asymmetric electrodes (grey arrows represent the direction of the n-DEP force). Reproduced with permission from [50] B. Çetin and D. Li, “Dielectrophoresis in microfluidics technology”, Electrophoresis, 2011.

Gallo-Villanueva et al. proposed a design comprising an array of cylindrical insulating posts to separate selectively or to concentrate a mixture of viable and non-viable microalgae [60]. The microalgal cells were separated with the DEP force experienced by living and non-living cells. The difference in the cell membrane conductivity of living and dead microalgal cells was responsible for the separation. Song et al. [61] designed an insulating microchannel with a triangular hurdle in the middle for continuous separation of microalgae by DC–DEP. Figure 3 represents the microfluidic microalgae sorting.

### 3.2. Inertial Microfluidic-Based Sorting

Inertial microfluidics are built on flow phenomena at intermediate Reynolds numbers (Re) (1 Re 100); Re is a dimensionless parameter characterizing the ratio of inertial and viscous forces in a flow. Inertia and fluid viscosity are finite in this regime and can give a deterministic character, allowing exact focusing and ordering of cells within a microchannel [62,63,64]. Inertial microfluidics have significantly increased sorting efficiency in separating microalgae samples from bacterial contamination [62]. 

## 4. Cell Transformation

This study focuses on microfluidic microalgal biotechnology-based biofuels. Single-cell resolution with high-throughput cell identification, cell separation, and efficient cell transformation are among the new developments. Strain selection and development, cell cultivation, and downstream processing are all topics of research [65].

The conditions for a microfluidic device for microelectroporation and microsonoporation in a flow-through scheme for highly efficient and high-throughput molecular delivery into mammalian cells are explained. The microfluidic electro-sonoporation device is a multi-modal cell portion device that simultaneously applies ultrasonic waves and electric fields [66].

### 4.1. Digital Microfluidic Platform

Do Jin Im and co-authors proposed a digital microfluidics-based electroporation system to manipulate droplets and to conduct gene transformation on applying an electric field generated with patterned electrodes [67]. In this system, two distinct electroporation mechanisms were accomplished. 

In the first mechanism, when a droplet comes into contact with the two electrodes, a direct current from the applied voltage runs through the droplet, resulting in static droplet electroporation. In the second mechanism, when only one electrode makes contact with the droplet, the droplet moves across the electrode pattern, causing electroporation. This pattern has shown a promising observation due to its ability to interact with other tasks such as in-droplet cell culture.

### 4.2. Droplet Microfluidics-Based Designs

**Figure 4:**
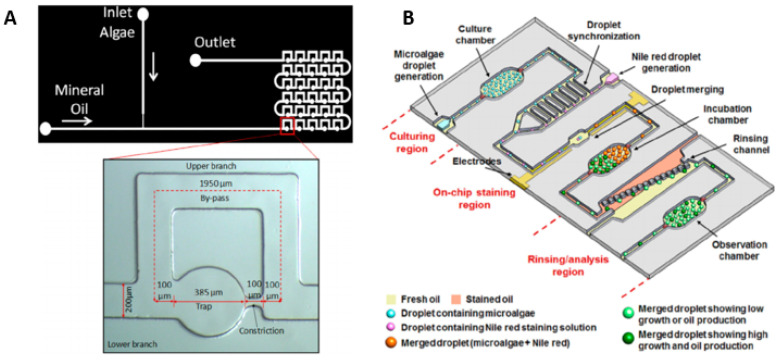
High-throughput droplet or digital microfluidics platform for microalgae culture and analysis. (**A**) Schematic diagram of the PDMS microfluidic device and the magnified image of a loop highlighting the various geometric dimensions. Reproduced with permission from [69] A. Dewan et al., “Growth kinetics of microalgae in microfluidic static droplet arrays”, Biotechnology and bioengineering, 2012. (**B**) A droplet microfluidics platform for rapid microalgal growth and lipid production analysis. Reproduced with permission from [70] H. S. Kim et al., “A droplet microfluidics platform for rapid microalgal growth and oil production analysis”, Biotechnology and bioengineering, 2016.

Modifying the properties of microalgae to possess desired traits has been a subject of interest [63,64], as it makes possible the delivery of a target gene into cells. Some commonly used methods are called electroporation or vortexing with glass beads [67,68], but these traditional methods are inefficient because of the thick cell walls of microalgae. Qu et al. reported a droplet microfluidic-based continuous-flow electroporation method that addressed problems faced during the exogenous DNS transfer. The method eliminated the random gene diffusion problem, which is commonly observed. During the electroporation process, there is also a chance of damaging foreign DNA being delivered into the cell. The study of spatially constrained droplets with droplet microfluidics was used to encapsulate microalgal cells and target DNA. For droplets having cells and DNA flow through five pairs of microelectrodes biased with a constant electroporation voltage, in-droplet electroporation occurred. The transformation efficiency of wild-type *C. reinhardtii* CC-124 cells employing this approach was 200 times that of the common bulk process. Figure 4 shows some digital microfluidic platforms for microalgae culturing.

## 5. Fuel Cell

The cost and demand of non-renewable resources for energy supply can be decreased on using more renewable energy, increasing competition and diversifying our energy supplies. The next generation of sustainable energy could come from microorganisms [71]. Microbial fuel cells (MFC) are devices in which living microbes are used to stimulate organic fuels into electricity conversion. These devices have recently attracted extensive attention as sustainable bioenergy systems [69,72]. MFC are driven by living microorganisms with a clean and sustainable feature. These fuel cells represent an emerging technique to generate electricity from renewable biomass [71]. Algae have been used as the anodic fuel in MFC for the production of electricity. Microalgae are essential for green energy resources. With great efficiency of fixing carbon dioxide, microalgae are broadly applied for biofuel production [70,73]. Microalgae are an important source of biofuel production due to competitive advantages such as not requiring access to a global food supply chain, high energy density, and the ability to absorb carbon dioxide to reduce global warming [74]. As oil-containing crops require large land areas, microalgae become a promising feedstock for biodiesel production. Microalgae have a more significant growth rate and lipid content than crops [73].

The generation of bioelectricity from MFC can be from heterotrophic and photosynthetic microorganisms or both in a single MFC system. Heterotrophic MFC (or simply called MFC) harvest electrical power by metabolic products of microbial respiration. The MFC need a continuous supply of organic carbon as energy. Photosynthetic MFC (or bio-solar cells) generate bioelectric power using bio-catalytic reactions of the photosynthetic microorganisms, such as cyanobacteria or algae. Hybrid MFC are the collective name for a new biofuel cell system that integrates both heterotrophic and photosynthetic microorganisms to generate bioelectricity [71].

The paper shows fuel cells of two types: a miniature microbial fuel cell (μMFC) and a small photosynthetic electrochemical cell (μPEC). This paper used a bulk silicon micromachining technique to fabricate the anode and cathode compartments of fuel cells for μMFC and μPEC. A μPEC explored blue-green algae for electricity generation under light. When the light was turned off, the μPEC functioned as a μMFC. In the anode, two distinct microorganisms were used as biocatalysts. (1) *Saccharomyces cerevisiae* (baker’s yeast) catalysed glucose, and (2) *Phylum cyanophyta* (blue-green algae) produced electrons in a photosynthetic reaction under light. The PEC continued to generate power in the dark by utilising glucose created under light [75].

**Figure 5:**
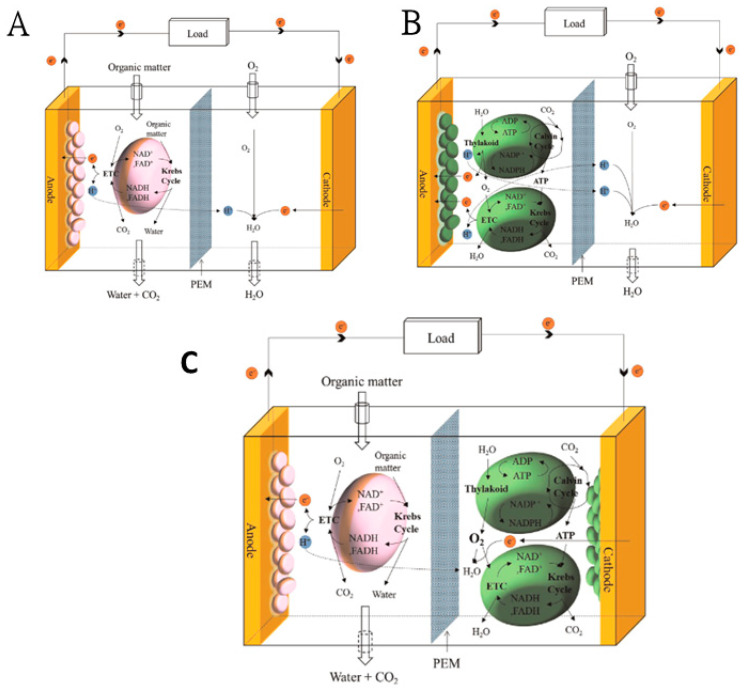
Principle of operations in an MFC. (**A**) Working principle of heterotrophic MFC. (**B**) Working principle of photosynthetic MFC. (**C**) Working principle of hybrid MFC. Reproduced with permission from [71] S. Choi, “Microscale microbial fuel cells: Advances and challenges”, Biosensors and Bioelectronics, 2015.

Bio-electricity production from phytoplankton, *Chlorella vulgaris*, and a macrophyte, *Ulva lactuca*, was examined in single-chamber microbial fuel cells (MFC). MFC were fed with the two algae in powdered form. The differences in energy recovery, degradation efficiency and power densities demonstrated that algae can serve as a renewable source of electricity production in MFC [76]. A micro-photosynthetic power cell (μPSC) as an energy source device from photosynthesis of blue-green algae with a polymer-based photosynthetic power cell was explained. Under test conditions, the proposed μPSC device produced a power density of 36.23 μW/cm^2^, voltage density of 80 mV/cm^2^ and current density of 93.38 μA/cm^2^ under test conditions [77]. Figure 5 shows various schematic MFC diagrams.

Microfluidics are used to control and manipulate almost picolitres of liquid with micrometre channel dimension. The precise control of microfluidic bioreactor devices extends their use for biofuel production. The microfluidic systems having bioreactors are comparable with the physical dimensions of cells and microorganisms. For this reason, microfluidic bioreactors are ideal for study of the behaviour of fuel-producing cells and their characteristics in the microenvironment. The positive attributes of microfluidics have to be envisaged to integrate the entire laboratory (lab-on-a-chip), automate genetic engineering and perform all functions in a single chip or device [77].

The current microfluidic techniques for microalgal downstream treatment in biorefinery and biofuel industries can be classified into biomass concentration, extraction of cellular content and biomass transformation. The downstream device fabrication requires specific materials able to resist extreme pressure, temperature and solvent [78].

The optimization of biofuel production is controllable with a single-chip system made as a miniaturized bioreactor. This flow rate, temperature and oxygen of the system can be monitored and controlled with these microfluidic devices [79]. Table 1 shows a comprehensive summary of different microfluidics operations of various species based on their operating mechanism.

## 6. Other Applications of Microfluidic Technology

### 6.1. Metabolic Engineering

Microfluidic systems with microalgae of desired traits can be generated efficiently through genetic and metabolic engineering. Gene assembly in robotic technology requires considerable operational cost. The microalgal strains were discovered from the local environments chosen for the production in particular geographical regions, as they typically express superior adaptability to the local environmental conditions. Native microalgae can be utilized as indicators for the application of various environmental monitoring [65].

### 6.2. Light-Controllable Photobioreactor

Microfluidic platforms have shown advantages for investigating the best conditions for microalgae production because of their great throughput and requirement of small amounts of sample or reagent. Lighting conditions such as intensity, duty cycle, spectral composition, pH, temperature, salt, CO_2_ and nutrient concentrations are some culture parameters studied using the microfluidic platforms. As the volume of microalgae culture in a microfluidic system is small, light self-shading effects are minimal, which allow for the collection of detailed information on cellular responses to illumination conditions. As a result, in the past three years, the use of microfluidic systems to optimize lighting settings has increased rapidly. Previously, Wang et al. [80] mentioned detailed information for the design and fabrication parameters of a microfluidic photobioreactor. The simplest method to provide the varied lighting conditions to a microfluidic device is to place the entire system in a light-controlled environment [81,82]. As most microalgal culture microfluidic devices are made of transparent materials such as PDMS and glass slides, the light intensity inside the microfluidic chambers is identical to the exposure intensity, although PDMS has identical refraction indexes for varied visible light wavelengths that can cause light dispersion [83]. Various filters have been employed to enable varied light intensity conditions on a single device [84], or an additional microfluidic layer containing fluids with varied concentrations of black dye [85] can be employed on top of the culture chambers. A simple LED array [21] or LED display with multiple LED backlights arranged in an array [86] is applicable for more sophisticated variations of lighting conditions. The LED array consists of diodes with fixed emission wavelengths and duty cycles that are easily adjustable. This device was used to study the growth and lipid production of *Cyclotella cryptica* algal species; their study showed that blue light (λ = 450 nm) increased growth, whereas yellow light (λ = 580 nm) increased the lipid content in *C. cryptica*. Their microfluidic system also allowed them to observe the relation between light wavelength and reactive oxygen species (ROS) for study of the wavelength-dependent accumulation of lipids. The results indicate that lipid accumulation is highly related to ROS amount increment, but it is possible that *C. cryptica*, under yellow light, could not produce antioxidants to cope with oxidative stress and thereby accumulate more lipids. A programmable LED display with multiple LED backlights arranged in an array provided more flexible adjustments for the sophisticated adjustment of light wavelength and intensity. In the microfluidic system developed by Luke et al. [87], about thirty combinations of spectral compositions and light intensities were tested. Their results indicated that *Synechococcus elongatus* showed the greatest growth at light intensity 42 μmol·m^−2^ s^−1^, with a spectral composition of ~90% red hue, which is the ratio between the red and full spectrum. The optimal light intensities leading to the greatest growth rates were found to vary significantly (42–360 µmol·m^−2^ s^−1^) depending on the tests. The variations were most likely due to the varied microalgae strains and their spectral characteristics. Furthermore, when other metabolites were required, the optimal lighting conditions varied. For example, the lipid content of *Neochloris oleoabundans* [81] and astaxanthin in *Haematococcus Pluvialis* [82] in the same microfluidic bioreactor required different lighting conditions.

### 6.3. Gradient Generators

As a continuous supply of fresh medium is required for microalgae culture, gradient-based microfluidic devices can be used, consisting of a mechanical trap for microalgae cultivation. The growth of *Chlamydomonas reinhardtii* was investigated by Eu et al. [88]. Holcomb et al. [89] used a TAP medium (nitrogen-depleted medium, Ca^2^-depleted, and herbicide (methyl viologen) added) in which sodium acetate was serially diluted with eight concentrations between 0 and 10 g L^−1^ [90] to find the optimal concentration for algal growth (5.72 g L^−1^) and lipid content (10.00 g L^−1^). Multiplexed results were also obtained from five trapping chambers having the same acetate concentrations. In each device, forty tests were conducted. Similarly, a device developed by Zheng et al. [91] for toxicity assessments of five microalgae strains utilized eight cultivation chambers with gradients of copper concentration, which ranged from 0 to 40 µmol L^−1^ with an exposure of 72 h. Batch and chemostat assays were performed. Their work indicated that, in *Chlorella* sp., chlorophyll fluorescence decreased with copper concentration, whereas in *Phaeodactylum tricornutum*, it increased.

## 7. Discussion

The production cost of biofuels from microalgae is large; a profit can hence be achieved on exploiting the entire ingredients in microalgae. This factor makes research related to producing biofuels from microalgae still zealous to pursue. To ensure successful and economically viable algae-based biofuel production, a critical factor is to perform effective and efficient screening of algal strains with high lipid content, high growth rate and high tolerance to environmental parameters during the bioprospecting stage [74].

Recent breakthroughs in electro-microbiology systems resulting from research on microbial fuel cells (MFC) are gaining traction as a potential “green” energy technology and energy-efficient wastewater treatment solution. Microscale MFCs are gaining popularity in a variety of fields, including analytical study tools, energy storage devices, and toxicity biosensors [71]. Microfluidic systems, in general, have a higher potential for massively parallel analysis than traditional methods. Droplet microfluidic devices have been used to perform various high-throughput screening experiments. Similarly, microalgae sensing using microfluidic systems incorporating electrical or optical approaches ensures label-free, noninvasive and sensing in situ for diverse biomolecules. An extensive study of chemical and biological sensing of microalgae-utilizing droplet microfluidics combined with micro-laser is required. Microfluidic bioreactors can help to examine microalgae growth behaviour. Microfluidics allow for the control of critical characteristics such as temperature, light and so on, which are crucial for the growth and health of microalgae.

Microfluidic flow cytometry has the potential to be a viable alternative technology because of its compactness and portability, which enables microalgae analysis on site and classifications during bioprospecting. Various microfluidic flow cytometry devices are discussed to be capable of monitoring chlorophyll autofluorescence of cells flowing across the optical detection zone in a microfluidic channel, which could analyse and distinguish microalgal cells. Microfluidic photo bioreactor devices are also discussed, which provide varied lighting conditions to a microfluidic device on placing the entire system in a light-controlled environment. Gradient-based microfluidic devices can be seen, which can be used to provide varied medium concentrations for specific requirements to study their importance on algal growth and metabolite generation.

Overall, microfluidic systems are suitable technological platforms because they offer numerous benefits such as rapid analysis, great precision, portability and the potential to integrate various functionalities on a single chip.

## 8. Conclusions and Future Scope

Thus far, few studies involving high-throughput microalgae screening have been reported, but as various microfluidic platforms mature and become easier to use, we expect that more research will be conducted utilizing such microfluidic platforms, potentially advancing the field significantly. Little research has been conducted on integrating microfluidic devices with traditional analytical instruments for improved throughput. There is a demand for such innovative microscale analytical tools or interfaces. Despite extensive research on cell sorting, harvesting, culture and lipid extraction, microfluidic devices continue to confront cost-efficiency challenges compared with traditional procedures. The number of commercialized approaches is still limited. One area that should be investigated and that can lead to some intriguing results is examining data obtained from microfluidic systems, which might provide foresight for large-scale and eventually industrial-scale culturing systems. Microfluidics possesses many advantages such as rapid analysis, great sensitivity and portability, and a capability to integrate multiple functions; it presents itself as a suitable technique to resolve issues encountered in microalgae biotechnology. There have been many applications, such as algal strain selection as per requirement, but most can be utilized for the downstream processes. A microalgal application such as biofuel generation requires, however, greater algal mass; for these processes, microfluidic devices are disadvantageous, as they require multiple integrations of various microfluidic devices to be used simultaneously, making the process costly and complicated.

To boost microalgae productivity, more improved culturing techniques need be developed, as well as novel biotechnologies such as gene editing. Microalgae can help to reduce CO_2_ levels in the atmosphere and purify wastewater. By overcoming hurdles and limits, algal fuel and bio-product technologies can be upgraded from the pilot to commercial scale.

## Figures and Tables

**Table 1:** Summary of microfluidic operations with their respective parameters.

Microfluidic Application/ Operation	Parameter/Species	Operating Mechanism	Throughput	Reference
Heterotrophic MFC	Consortia containing *Pseudomonas aeruginosa*, *Enterococcus faecium* and *Rhodoferax ferrireducens*	Extracellular electron transfer (EET)	-	[71]
Photosynthetic MFC	cyanobacteria or algae		-	[71]
Hybrid MFC	Heterotrophic and photosynthetic microorganism		-	[71]
Identification of Cell size	*Cyanothece aeruginosa*, *Scenedesmus acuminatus*, *Chlorella vulgaris*, *Microcystis viridis*, *Anabaenopsis* sp., *Navicula pelliculosa*, *Pseudokirchneriella subcapitata*, *Pseudana-baena* sp., *Monoraphidium griffithii*	Optical sensing	-	[28]
Cell Separation	*Chlamydomonas reinhardti*	Dielectrophoresis	-	[55]
Cell Sorting	*Chlamydomonas reinhardtii*	Dielectrophoresis	88.8%	[55]
Cell Purification	*Coenochloris signiensis*	Inertial focusing	97.3%	[55]
Cell viability	*Karenia mikimotoi Hansen*, *Chlorella vulgaris*, *N. closterium*, *Platymonas subcordiformis*, *P. delicatula*, *Dunaliella salina*	Optical sensing	~10 cells/min	[30]
Cell viability	*Dunaliella salina*	Capacitance-based sensing	~40 cells/min	[32]
Screening/sorting by lipid content	*Chlorella vulgaris*	Dielectrophoresis	~10^4^ cells	[51]
Screening/sorting by lipid content	*Chlorella vulgaris*	Dielectrophoresis, hydrodynamic flow	~100 cells/min	[53]
Size based sorting/screening	*Chlorella vulgaris*, *Pseudokirchneriella subcapitata*	Electro-osmotic field	30–40 cells/min	[59]

## Data Availability

Not applicable.
